# *De Novo* Assembly and Transcriptome Analysis of Bulb Onion (*Allium cepa* L.) during Cold Acclimation Using Contrasting Genotypes

**DOI:** 10.1371/journal.pone.0161987

**Published:** 2016-09-14

**Authors:** Jeongsukhyeon Han, Senthil Kumar Thamilarasan, Sathishkumar Natarajan, Jong-In Park, Mi-Young Chung, Ill-Sup Nou

**Affiliations:** 1 Department of Horticulture, Sunchon National University, Suncheon, Jeonnam, Republic of Korea; 2 Department of Agricultural Education, Sunchon National University, Suncheon, Jeonnam, Republic of Korea; Youngstown State University, UNITED STATES

## Abstract

Bulb onion (*Allium cepa*) is the second most widely cultivated and consumed vegetable crop in the world. During winter, cold injury can limit the production of bulb onion. Genomic resources available for bulb onion are still very limited. To date, no studies on heritably durable cold and freezing tolerance have been carried out in bulb onion genotypes. We applied high-throughput sequencing technology to cold (2°C), freezing (-5 and -15°C), and control (25°C)-treated samples of cold tolerant (CT) and cold susceptible (CS) genotypes of *A*. *cepa* lines. A total of 452 million paired-end reads were *de novo* assembled into 54,047 genes with an average length of 1,331 bp. Based on similarity searches, these genes were aligned with entries in the public non-redundant (nr) database, as well as KEGG and COG database. Differentially expressed genes (DEGs) were identified using log_10_ values with the FPKM method. Among 5,167DEGs, 491 genes were differentially expressed at freezing temperature compared to the control temperature in both CT and CS libraries. The DEG results were validated with qRT-PCR. We performed GO and KEGG pathway enrichment analyses of all DEGs and iPath interactive analysis found 31 pathways including those related to metabolism of carbohydrate, nucleotide, energy, cofactors and vitamins, other amino acids and xenobiotics biodegradation. Furthermore, a large number of molecular markers were identified from the assembled genes, including simple sequence repeats (SSRs) 4,437 and SNP substitutions of transition and transversion types of CT and CS. Our study is the first to provide a transcriptome sequence resource for *Allium* spp. with regard to cold and freezing stress. We identified a large set of genes and determined their DEG profiles under cold and freezing conditions using two different genotypes. These data represent a valuable resource for genetic and genomic studies of *Allium* spp.

## Introduction

Cold and freezing stresses are common environmental abiotic stresses. Plants undergo a variety of physiological and biochemical changes as defense systems for withstanding cold (0–15°C) and freezing (<0°C) conditions. Although not all plants can tolerate cold and freezing temperatures, many plants show tolerance to cold and freezing via a phenomenon termed ‘cold acclimation’ (CA) [1, 2]. During cold acclimation, many genes show differential expression, responding to cold and freezing stress at the transcriptional level [3, 4]. For example, in the model plant *Arabidopsis thaliana*, many transcription factors and thousands of genes are thought to be involved in cold stress responses [4]. In general, plants from temperate regions are considered to be chilling tolerant to varying degrees and their freezing tolerance is increased by previous exposure to cold and freezing temperatures [5]. Recent studies have aimed to analyze the functions of stress-inducible genes, not only to understand the mechanism of cold and freezing stress responses, but also to improve the stress tolerance of other plants by gene transfer. The CBF/DREB (C-repeat [CRT]/dehydration responsive element [DRE] binding factor) transcription factors mediate a cold acclimation pathway that induces transcriptional reprogramming of cold regulated (COR) genes [1, 2]. CBF, an AP2/ERF family transcription factor, binds to and induces a number of COR genes (CBF regulon) that contain a CRT/DRE motif (CCGAC) in their promoters, thereby conferring freezing tolerance [6–11].

Bulb onion (*Allium cepa*) is a major vegetable crop that belongs to the *Amaryllidaceae* family, including 300 species of which only 70 have been cultivated [12]. In onion, the pungency level is related to the content of polyphenols, vitamins and sulphur-containing compounds [13–15]. Those compounds also affect various aspects of tolerance of environmental stresses, including cold and freezing stress. In spite of the status of *Allium* species as a major vegetable crop with both nutritional and medicinal value, genetic research in *Allium* has lagged behind that in other vegetable crops and little genomic information is available, in part because of the enormous size of the *Allium* genome (16.3 Gb) [16, 17]. At present, research examining stress-inducible genes of *A*. *cepa* has focused on physiology and identification of specific response genes. However, to our knowledge, no study involving individual or large-scale screening of cold and freezing stress-related genes and molecular marker identification has been published to date. Next generation sequencing (NGS) has enabled large-scale transcriptome data analyses that have dramatically improved the efficiency of gene discovery at the genome level without prior knowledge of reference genome sequences. Since the first use of Solexa/Illumina’s Digital Gene Expression (DGE) system to study the zebrafish transcriptome after *Mycobacterium marinum* infection [18], RNA-Seq and DGE technology have been widely used to identify plant genes related to important agronomic traits, including those expressed under stress conditions [19, 20]. Large portions of the transcriptomes of *A*. *thaliana* and *Brassica rapa* are regulated by abiotic stresses such as cold, salt and drought [4, 21]. In wheat, cold stress induces transcriptome reprogramming, with over 2% of the wheat genome showing a greater than two-fold change in expression [22]. However, transcriptome changes and biochemical functions of cold and freezing stress-regulated genes remain unknown. Molecular markers play a crucial role in applications related to genetic diversity in plant breeding. Many EST-SSR markers have been developed using high-throughput sequencing data, for instance 39,257 EST-SSRs from healthy rubber trees and 4,609 EST-SSRs from date palm EST sequences; in both cases, the primers were validated to have polymorphism in different cultivars [23, 24].

In this study, we obtained transcriptomes from contrasting lines of *Allium cepa* treated with cold (2°C), freezing (-5, -15°C) and control temperature (25°C) using Illumina paired-end sequencing technology. The resulting sequence data were *de novo* assembled and annotated without prior reference genome information. Furthermore, we compared the gene expression profiles between treated and control plants, and SSRs and SNPs markers were predicted *in silico*. To our knowledge this is the first report on the cold and freezing transcriptome of bulb onion. Hence, this research significantly enhances our understanding of the transcriptional changes underlying the cold and freezing response in *A*. *cepa*. Our study provides information towards elucidating cold and freezing tolerance mechanisms; in addition, the molecular markers predicted and developed herein should facilitate gene mapping and genetic diversity analysis.

## Materials and Methods

### Plant materials and electrolyte leakage test

Two bulb onion (*Allium cepa*) lines that exhibit contrasting sensitivity to cold stress, cold tolerant (CT) 36122 and cold susceptible (CS) 36101, inbred lines were collected from the Nongwoo Bio Seed Company, Suwon, Korea. The seeds were grown under controlled conditions in a growth chamber for ~4 months at 25°C with 16 h light/8 h dark photo period. For cold and freezing stress treatment 25°C (control) plants were exposed at 24 h to 2°C, -5°C, and -15°C. After treatment, the 4^th^ or 5^th^ leaf of CT and CS were collected. Immediately after treatment, electrolyte leakage from the cold and freezing-stressed and control plant samples was measured using previously described methods [25, 26], with some modifications. Briefly, 10 leaf discs (1 cm in diameter) excised from fully expanded leaves of 3–4 plants were placed in a glass tube with 10 ml distilled water, and incubated on an orbital shaker at 150 rpm for 30 min at room temperature. The initial conductivity (I) was measured using a CON110 conductivity meter (Oakton Ins. USA). The leaf discs were then kept in a boiling water bath for 10 min and cooled to room temperature before the final conductivity (F) was measured. The relative electrolyte leakage was calculated using the formula I/F X 100. Significance tests were carried out using Least Significant Difference (LSD) at P = 0.05 level was used to compare the means.

### RNA extraction and library preparation

The total RNA was isolated using TRIzol reagent (Invitrogen, USA) according to the manufacturer’s instructions. Poly (A)+ mRNA was purified with oligo (dT) beads, and then the mRNA was randomly segmented into small fragments by divalent cations (Fragmentation Buffer, Illumina, Hayward, CA) at 94°C for 5 min. These short fragments were used as templates to synthesize the first-strand cDNA using random hexamer primers. The second-strand cDNA was synthesized using RNaseH and DNA polymerase I. Short cDNA fragments were purified with the QiaQuick PCR extraction kit. After that, the cDNA fragments were connected with sequencing adapters according to Illumina’s protocol (San Diego, CA, USA). For each sample, at least ~20 μg total RNAs was used for RNA-sequencing at the Beijing Genomics Institutes (BGI)-Shenzhen, (Shenzhen, China). For the reverse transcription-polymerase chain reaction real-time PCR experiments, the total RNA was prepared following the aforementioned procedures.

### Sequencing, *de novo* assembly and functional annotation

Individual cDNA samples of CT and CS treated at 2°C, -5°C, -15°C and followed by 25°C (control) were prepared for sequencing. The constructed paired-end (PE) sequence libraries of each sample were sequenced by Illumina HiSeq 2000 sequences (2 × 100 bp read length). The generated raw read quality was assessed using FASTX-Toolkit (http://hannonlab.cshl.edu/fastx_toolkit/). To obtain high-quality sequencing data, adaptor sequences and low-quality reads were removed using ‘sickle' (https://github.com/najoshi/sickle) and ‘SeqPrep’ (https://github.com/jstjohn/SeqPrep), respectively, with the default parameters. We calculated Q20, Q30 and GC contents for all cleaned data for each sample. Further, poly-N and low-quality reads were removed and high-quality cleaned data were subjected to all downstream calculations. The *de novo* assembly program Trinity (http://trinityrnaseq.sourceforge.net/) was used for short read assembly and to calculate the N50 number. We used the pipeline of ‘transcript_to_best_scoring_ORFs.pl’ pipeline in trinity software to predict the ORFs with default parameters [27]. The raw sequence data obtained by PE RNA-sequencing have been deposited in the NCBI SRA and TSA (National Centre for Biotechnology Information Short Read Archive, http://www.ncbi.nlm.nih.gov/sra and Transcript Shotgun Assembly, http://www.ncbi.nlm.nih.gov/genbank/tsa/) database under accession number **SRP064878** and **GETF00000000**, respectively.

### Functional annotation and classification

For functional annotation, the assembled genes that might putatively encode proteins were searched against the nr (BLASTX) (NCBI non-redundant protein sequences, http://www.ncbi.nlm.nih.gov/), Swiss-Prot (http://www.expasy.ch/sprot/), and nt (NCBI non-redundant nucleotide sequences) databases using Blast2GO with minimum E-value of <1e^-10^ as the threshold [28]. With nr annotations, the Blast2GO program was used to assign GO annotations according to component function, biological process and cellular component categories [29]. In a final step, COG (http://www.ncbi.nlm.nih.gov/cog/) [30] and KEGG (http://www.genome.jp/kegg/) [31] annotation was performed for all of the genes.

### Mapping and differentially expressed gene analysis

The procedure for constructing the DGE sequencing libraries was the same as that for constructing the transcriptome sequencing libraries. After the raw data were generated and the data-processing steps were completed, the clean reads were mapped to the assembly transcriptome reference sequences using RSEM with default parameters [32]. Gene expression levels were calculated based on the numbers of reads mapped to the reference sequence, using the FPKM [33] method. After calculating gene expression levels, the differentially expressed genes (DEGs) were screened by comparing gene expression levels. Differential expression analysis was performed with software ‘edgeR’. The resulting P values were adjusted using Benjamini and Hochberg’s approach for controlling the false discovery rate (FDR ≤ 0.01, log2FC ≥ 2).Functional enrichment analysis, including GO and KEGG analyses, was performed using genes of the entire transcriptome. For this analysis, a Bonferroni-corrected P-value ≤0.05 was used. GO functional enrichment analysis was carried out using Goa tools (https://github.com/tanghaibao/Goatools).

### *In silico* analysis of molecular marker

The detection of SSRs from the total of assembled genes was performed using Msatcommnader software [34]. The software accepts FASTA formatted sequence files, the sequence ID, SSR motif, number of repeats (di-, tri-, tetra,-penta- or hexa-nucleotide repeat units), GATK (Genome analysis tool kit) software was used to perform SNP calling with default parameters [35]. Along with the SNP and position, additional information about the called SNPs, such as SNP quality, depth of coverage.

### Quantitative real-time PCR validation

To confirm the DEG results, quantitative real-time reverse transcription PCR (qRT-PCR) was performed. Twenty-five genes were chosen based on FDR≤0.005 value to increase the reliability of DEGs for qRT-PCR analysis in the CT vs. CT libraries. Gene-specific primers were designed based on the sequencing results using Primer 3 (version 6.0) [36] (S1 Table). The real-time PCR was performed on an Illumina Eco real-time machine (PhileKorea Technology, Korea) using 1 μl first-strand cDNAs mixed with SYBR Premix Ex TaqTM (Toyobo, Japan). The thermal cycling conditions were 30 s at 95°C, followed by 40 cycles of 5 s at 95°C and 31 s at 58°C. All reactions were performed in triplicate. The relative expression levels of each gene were calculated using the 2^-ΔΔCT^ method normalized to the internal control gene, *Allium* ssp. *Actin*.

## Results and Discussion

### Illumina paired-end sequencing and *de novo* assembly

Two well-known contrasting genotypes of Korean onion lines were chosen in this study. Cultivars 36101 and 36122, which are widely adapted to winter and spring/summer seasonal cultivation in South Korea, respectively, were use as cold susceptible (CS) and cold tolerant lines (CT),respectively [37]. To precisely evaluate their tolerance of cold and freezing stress, we compared the electrolyte leakage test of leaf samples after treatment for 24h with different temperature that were further used for transcriptome experiments. Electrolyte leakage is inversely proportion to freezing tolerance [38–40], and there great differences between the two lines during cold (2°C) and freezing stress (-5°C) (S1 Fig). Comparison of control 25°C and -5°C observations revealed that the tolerant line had adapted to the freezing stress and was able to protect the membrane of from further damage. Two way ANOVA statistical analysis showed that observation of electrolyte leakage was significantly different at different temperatures within and between genotypes.

To identify any freezing-tolerance genes induced to create this phenotypic response in onion, total RNA was isolated from plants treated with control temperature (25°C) or 2, -5 and -15°C. Paired-end RNA-sequencing using the Illumina HiSeq 2000 platform was performed with control and treated samples. A total of 452 million paired-end reads were produced for control and treated samples (Table 1). Clean reads were obtained from paired-end reads by performing high stringency filtering with default parameters to remove adaptor and/or vector read sequences. Ultimately, 450,821,674 (99.6%) pooled high-quality reads were used for *de novo* transcriptome assembly. All clean reads were also deposited in the National Center for Biotechnology Information (NCBI) and can be accessed in the sequence read archive (SRA) under the accession number **SRP064878**. With the Trinity program [41], 93,637 transcripts were generated with an average length of 1,331 bp and an N50 length of 1,625 bp; the maximal transcript length was 15,559. These results were comparable to those for other transcriptome analyses in plants, such as *H*. *ammodendron* (N50 = 1,345 bp, average length = 728bp, [19]) and *Ammopiptanthus mongolicus* (N50 = 1,343 bp, average length = 816 bp, [42]). These data indicate that the assembly was effective in capturing a large portion of the *A*. *cepa* transcriptome. The assembled transcripts were initially predicted to correspond to 54,047 genes, which were screened for best scoring ORFs predicted using the Trinity program, resulting in 50,280 genes as best candidate genes, were deposited into TSA (**GETF00000000**). The average length of these genes was 807bp, and their lengths ranged from 150bp to 14,970bp.

**Table 1:** Sequencing statistics of the *Allium cepa* L. transcriptomes.

Summary	Total nucleotides
Total number of reads	452,194,370
Clean reads	450,821,674
Avg. of GC %	42.4
Total no. of isogenes/transcripts	93,637
Avg. length in bp	1,331
Minimum length in bp	500
Maximum length in bp	15,559
N50 length	1,625
Total no. of genes	54,047
Avg. length in bp	807
Minimum length in bp	150
Maximum length in bp	14,970

### Functional annotation and classification

**Fig 1:**
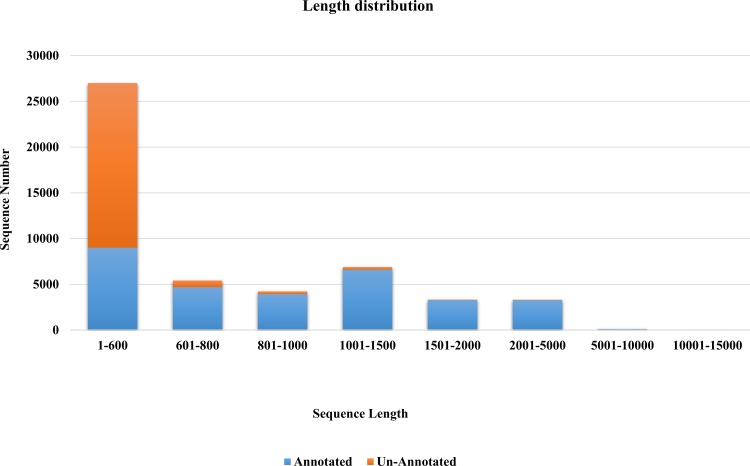
Histogram of sequence length distribution and number of sequences annotated. Assembled genes were searched in the nr protein database using BLASTX with a cut-off E-value 1e^-10^.

To identify their putative functions, the assembled genes were screened against public databases such as the NCBI non-redundant (nt, nr), Gene Ontology (GO), Kyoto Encyclopedia of Genes and Genomes (KEGG) and Cluster of Orthologous Groups (COG) databases using BLASTX with an E-value threshold of 1e^-10^ (default parameters). This analysis allowed good annotation of genes with more than 1,000bp, whereas 33.3% of sequences less than 600bp could be matched to known proteins (Fig 1). Among all of the genes, 30,926 (61.5%) showed significant similarity to entries in the nr database, similar to values reported for other *de novo* assemblies [19]. In BLAST searches against the NT database, a smaller percentage of similarities were found Table 2. In total, BLASTX searches against the nr database identified 19,354 accessions that did not match proteins from the public databases, suggesting that our Illumina paired-end sequencing included significant numbers of the cold and freezing stress-related genes in *A*. *cepa*. Among the BLAST results, the species that provided the most matches was *Vitis vinifera* (5,443) and the next closest species was *Theobroma cacao* (2,420) (S2 Fig).

**Table 2:** Summary of the functional annotation of assembled genes.

Public database	Number of genes	Percentage (%)
Annotated in NR	30,926	61.5
Annotated in NT	1,305	3.0
Annotated in GOs	23,446	46.6
Annotated in COG	15,602	31.0
Annotated in KOG	14,993	29.8
Annotated in KO/KEGG	9,656	19.2
Total genes	50,280	100

**Fig 2:**
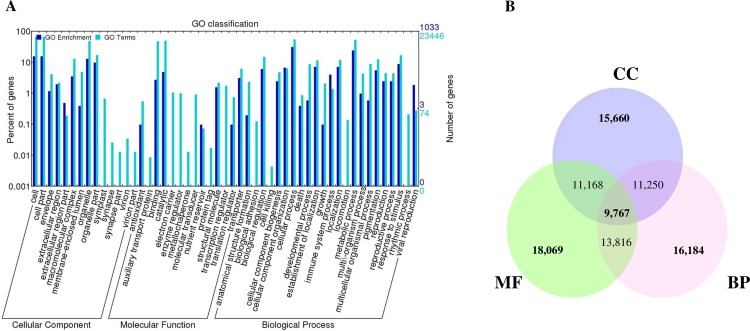
(A) Gene ontology classification of the assembled genes. Histogram of the GO annotation and enrichment analysis was generated by the BGI WEGO software. Genes were grouped into three main GO categories: Cellular component (CC), Molecular function (MF), Biological Process (BP). The right Y- axis indicates the number of genes in a category. The left Y-axis indicates the percentage in a specific category. One gene could be assigned with more than one GO term. Color indicates GO enrichment (significantly enriched GO terms with Bonferroni corrected p-value <0.05) and GO terms in all genes. (B) Venn diagram of assembled genes were accounted in three major categories of gene ontology classification.

To classify the functions of the predicted onion cold and freezing transcriptome genes, we used Gene Ontology (GO) analysis. Using the Blast2GO suite, 23,446 (46.6%) sequences were categorized into three different GO terms (Biological Process: BP, Molecular Function: MF and Cellular Component: CC) (Table 2). The three main categories were further classified into 58 functional groups compared with significantly enrichment analysis (Fig 2A and 2B). The most genes were classified under BP (60,578, 42.0%), followed by CC (57,202, 39.6%) and MF (26,316, 18.2%). Under the main three categories, seven groups of sub-categories were predominant in the GO classification. In the three largest categories of BP, ‘cellular process (GO: 0009987)’ and ‘metabolic process (GO: 0008152)’ were highly represented. Under the classification of MF, ‘catalytic activity (GO: 0003824)’ (11,715, 8.12%) and ‘binding (GO: 0005488)’ (11,300, 7.8%) were significantly represented, whereas other sub-categories contained only 3,301 genes representing 14.07% of the total genes. For cellular component, three sub-categories ‘cell (GO: 0005623)’, ‘cell parts (GO: 0044464)’ and ‘organelle (GO: 0043226)’ accounted for 68.2% of the genes. Fewer than half of the genes were not annotated in this study, likely due to the sequencing length or depth of coverage, as is common in studies performing *de novo* transcriptome analyses [43]. In addition, some of these genes might be unique to *A*. *cepa*.

**Fig 3:**
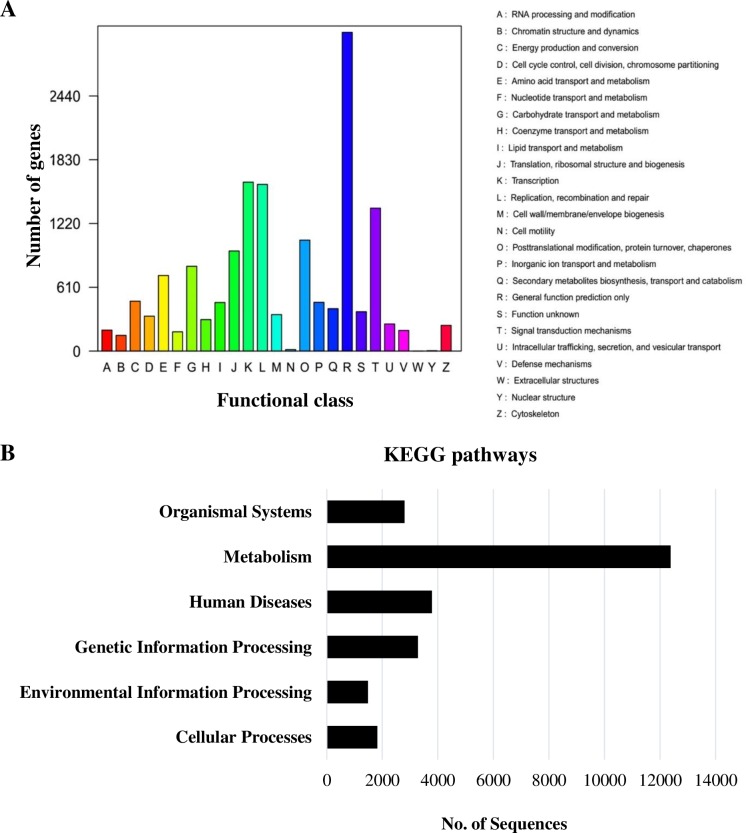
(A). Distribution of genes in the transcriptome with Clusters of Orthologous Groups (COG) functional classification. 10,549 genes were grouped into 25 categories. (B) Distribution of assembled genes in each KEGG pathway category. All genes were assigned under six major categories.

To further investigate their possible functions, we analyzed the assembled genes using the Clusters of Orthologous Groups (COG) database. Of the 15,602 genes with significant similarity to nr database proteins in this study, 10,549 genes were grouped into 25 functional classifications (Fig 3A). The clusters related to ‘general function prediction only’ (3,049, 19.54%) were the largest group, followed by ‘transcription’ (1,616, 10.35%), ‘replication, recombination and repair’ (1,594, 10.21%), and signal transduction mechanisms (1,367, 8.76%).

We further characterized the transcriptome data using pathway analysis with the Kyoto Encyclopedia of Genes and Genomes (KEGG) database. In total, 11,204 (22.28%) sequences were identified in six main categories that included 333 pathways (Fig 3B and S2 Table). ‘Metabolism’ was the category with the greatest number of genes (12,383, 48.4%), with metabolic pathways and biosynthesis of secondary metabolites as the two pathways represented by the most genes. Overall, these annotation and classification analyses add valuable information to the *A*. *cepa* transcriptome, with gene functions, annotation and metabolic pathways.

### Differentially expressed gene (DEG) library during cold and freezing stresses

**Fig 4:**
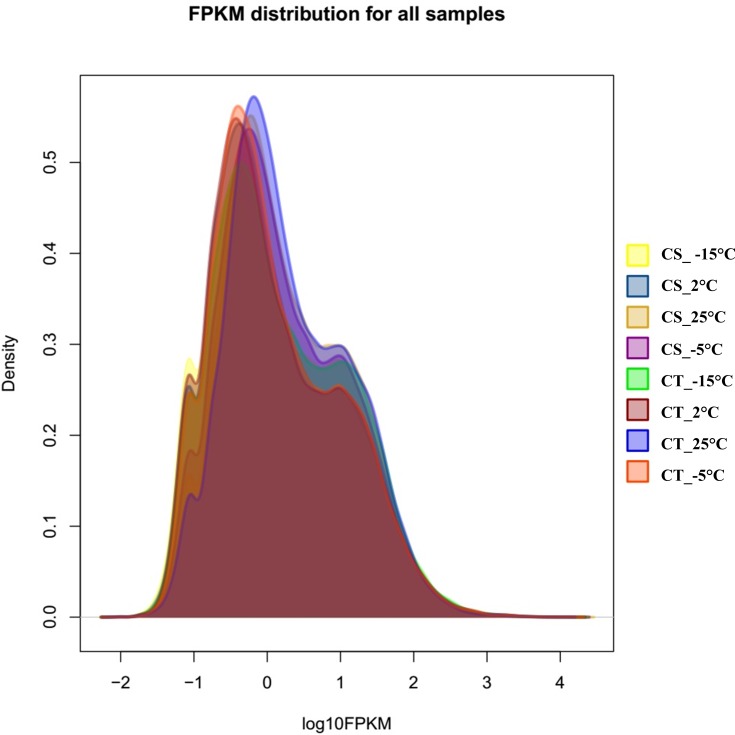
Density distribution of expression level based on log_10_ FPKM, exhibiting the overall differences in expression profile among the libraries.

To normalize the datasets for *de novo* transcriptome assemblies, we used RSEM software, which does not require reference genome and provides superior performance over other quantification methods [32]. The FPKM (Fragments per Kilobase per Million), TPM (transcripts per million) and EC (expected count) values for each mapped clean read were calculated (S3 Table). The obtained FPKM values from the libraries ranged from 0 to 15,935, and we visualized the density distribution of expression level in each DEG library based on the log_10_ value of FPKM (Fig 4). The distribution patterns of all libraries were overlaid with different peaks values. After normalizing the read density measurement to reveal significant responses to cold and freezing stress, genes were filtered by FDR (value ≤0.01) (False Discovery Rate) and a value of log02FC≥2 using edgeR software with default parameters. We performed comparative profile analysis using the aligned reads of CS vs. CT, and obtained 2,400 and 4,080 up- and down-regulated genes, respectively, which were commonly expressed in all of the profiles (S3 Fig). The results indicate that a large number of genes were involved in the cold acclimation process. Hence, we compared the two lines CS vs. CT lines during 25°C, 2°C, -5°C and -15°C treatments, finding that a total of 1,822, 2,921, 2,931 and 1,887 were upregulated and 1,786, 2,740, 2,665 and 2,182 were downregulated genes, respectively (S3 Fig). Among all the genes, 705 and 1,203 genes were upregulated and downregulated, respectively, in all the temperature profiles, identifying these 1,208 genes as candidate DEGs between the contrasting lines. However, 165 upregulated and 149 downregulated genes were predominantly expressed throughout the cold treatment at -5°C and also present in temperature profiles 2°C to -15°C between the two lines, whereas 346 upregulated and 334 downregulated genes were only among one of these three profiles. Genes that are commonly regulated across all the temperature treatments between the two lines might be candidate cold-responsive genes that alters plant tolerance during cold stress. Notably, -5°C freezing stress led to the greatest number of DEGs in CS and CT lines. Overall, 2,409 and 2,758 genes were differentially expressed in CS and CT lines, respectively, at 25°C vs. -5°C (S4A and S4B Fig). Of these DEGs, CS (712) and CT (1,258) were successfully annotated using the public databases. To explore the biological functions and correlation of these DEGs, we performed enrichment analysis of using GO terms of three major categories for the up- and down-regulated genes in the CS and CT profiles. The resultant GO terms enriched in CS and CT, were highly enriched terms are in BP ‘cellular process’ (GO: 0009987), in MF ‘catalytic activity’ (GO: 0003824), and in CC ‘cell’ and ‘cell part’ (GO: 0005623 and GO: 0044464, respectively) (S5 Fig).

### Metabolic pathway insights related to DEGs

**Fig 5:**
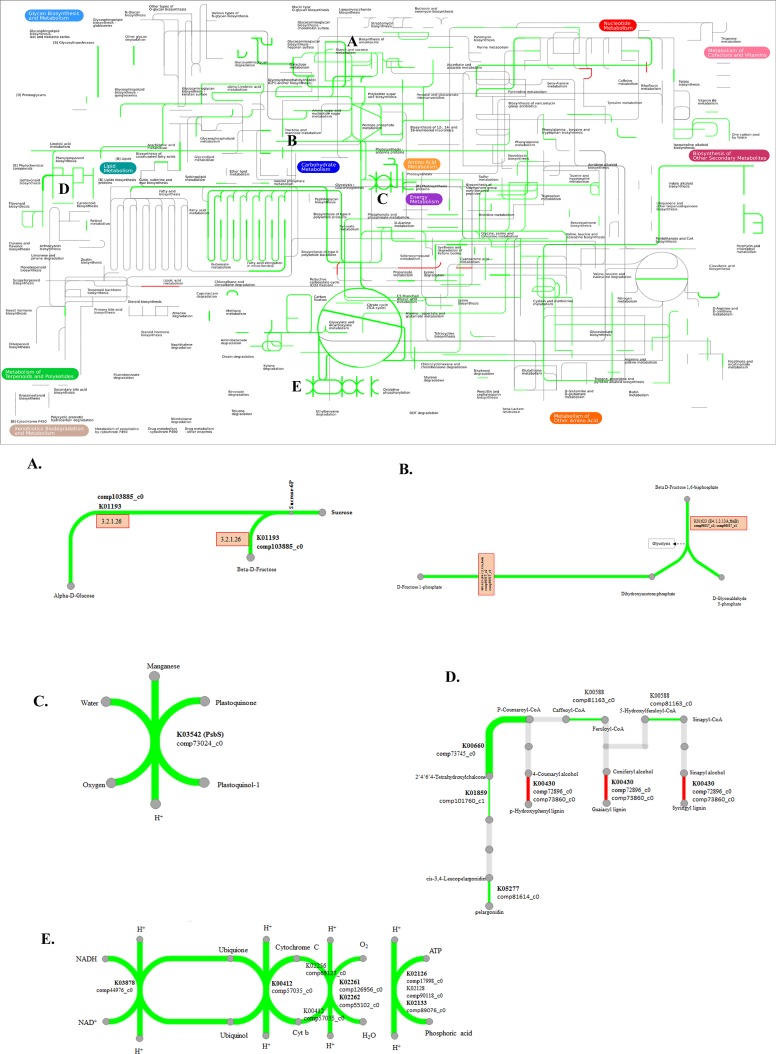
Metabolic pathways active during freezing temperature in onion. The red and green indicate up and down-regulated genes with tolerance and susceptibility, respectively. Major pathways of freezing-related metabolism is shown in (A) Starch and sucrose metabolism, (B) Fructose and mannose metabolism, (C) Energy metabolism, (D) Biosynthesis of secondary metabolites, (E) Oxidative phosphorylation.

For a global enriched pathway view of freezing metabolism, we used Interactive pathways (Ipath) explorer v2, which integrates 123 KEGG maps from 183 species [44]. Previous studies have effectively used RNA-Seq transcriptomics with interactive pathway analysis [45], for instance for a global view of Chinese bayberry metabolism analysis [46]. In this study, 491 genes were combined with DEGs of CS and CT profiles at 25 vs. -5°C (S4A and S4B Fig, S4 Table). In all, 88 KO (KEGG orthology) ids mapped to 31 metabolic pathways were identified (Fig 5). At -5°C, expression levels showed up- and down-regulation in the CS and CT profiles, and genes mapped to pathways including metabolism of carbohydrate, biosynthesis of other secondary metabolites, energy, cofactors and vitamins, other amino acids and xenobiotics biodegradation. In some pathways, metabolism of starch and sucrose and fructose and mannose enzymatically induced beta-d-fructose, alpha-d-glucose, and D-glyceraldehyde 3-phospate and D-fructose 1-phosphate, respectively (Fig 5A and 5B). More pathways showed differential expression during freezing stress conditions than under other temperatures, in accord with GO comparisons [47]. In energy metabolism, K03542 (Psbs) plastoquinone to plastoquinone-1 may be involved in photosystem II (PSII) intersystem electron transport chain; here, our expression data show that it might be related to attainment of a cold acclimated state and, hence, maximum freezing tolerance (Fig 5C) [48]. Fig 5D shows several pathways (K00588, K0060, K01859, K05277 and K00430) involved in biosynthesis of phenylpropaniod, flavonoid and peroxidase may change the composition of aromatic, pungency compounds during freezing stress. In oxidative phosphorylation, K02126, K02262 (O_2_ to H_2_O) acts as the primary terminal acceptor from PSI and PSII, and thereby leads to substantial generation of reactive oxygen species (ROS) and may also induce the rapid depletion of cellular damage (NAD/NADH) (Fig 5E) [49]. Overall, the results revealed interactive metabolic pathways associated with freezing tolerance gene expression during -5°C in CT and CS lines.

### Validation of differential gene expression

**Fig 6:**
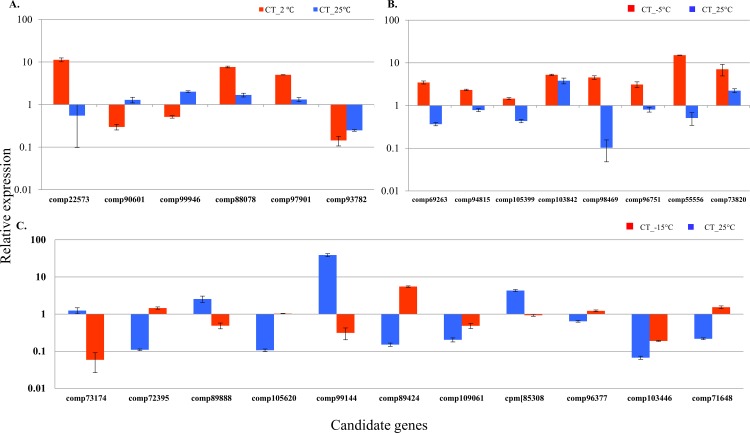
Validation of candidate DEGs during (A) cold, (B) cold & freezing, and (C) freezing stress using qRT-PCR. Error bars indicate standard deviation (SD). X-axis indicates, relative expression to the log2 fold change (log2FC), Y-axis indicates selected candidate genes of DEGs.

To further validate the gene expression profiles, we selected 25 candidate genes from the CT profiles of 25 vs. 2, -5, -15°C, for qRT-PCR (S1 Table). These genes included cold and freezing-associated genes such as those encoding ZAT12 [50], RAV [9], zinc finger [51], calcium binding protein, CAMK_KIN1 [52], AP2-like, DREB and UDP-forming [9]. All 25 genes were confirmed by qRT-PCR analysis to be differentially regulated by the respective temperature (Fig 6), although the fold changes were different from the transcriptome sequencing data. Based on annotation, information on gene function was available for all but two of these genes. The genes significantly induced by cold and freezing stress in *A*. *cepa* encoded products including 4 zinc finger proteins, 4 hypothetical proteins, UDP forming, CBL-interacting protein kinase, 2 heat shock protein, 2 CMLH and subset of transcription factors, 2 bHLH, AP2 domain containing protein, DREB-2, MYB2, and bZIP. During cold and freezing conditions, drastic changes occur in the transcript levels of genes that encode transcriptional factors, particularly those that function as activators, major TFs involved are DREB, MYB, CBL, bZIP, ZAT, HSPs, bHLH [7, 53, 54]. Apart from these TFs, other genes encode proteins such as chitinase and heat shock proteins that interacts with the anti-freeze related proteins, thus preventing oxidative stress and ice formation [55, 56]. Base on previous reports, the cold-inducible genes were analyzed using qRT-PCR and found to be expressed similarly as in transcriptome data with minor variations. During cold stress (-2°C), upon signal perception by plasma membrane, the signal is transduced into the nucleus by cascades of Ca^2+^ dependent kinases such as CIPKs (CBL interacting protein kinase) [57, 58] and they activates transcription factors and other proteins that maintain cell wall integrity, such as UDP proteins [59]. Colder temperatures (-5°C and -15°C) induces other defense-related genes that are triggered by different types of TFs. For instance, CBF family TFs control the downstream signaling cascade during cold and freezing stress [60]. Further, membrane proteins such as chitinase prevents oxidative stress by fortification of the cell membrane during cold and freezing stress. These membrane binding proteins encode an antifreeze proteins which inhibit the formation of ice in wheat [55]. In *Chenopodium album*, plastid or chloroplast small heat shock proteins confer tolerance during cold stress [56]. In *Arabidopsis*, zinc finger transcription factors increase tolerance by interacting with CBF family proteins [50]. Similarly in *Brassica rapa*, analyses using two cold genotypes they suggest bZIP are involved in cold tolerance [61]. Overall, our expression analysis of the 25 identified genes suggest that these genes are involved during cold and freezing tolerance and may work together to protect *A*. *cepa* from cold or freezing damage.

### *In silico* predictions of molecular markers

To enhance the quality of the sequence data assembly and to identify new molecular markers based on as SSRs (simple sequence repeats) and SNPs (single nucleotide polymorphisms) in onion, we screened the transcriptome gene dataset for SSRs using Msatcommander software with default parameters. A search for di-, tri-, tetra-, penta-, and hexa- nucleotide repeats yielded 1,258, 2,800, 301, 51 and 27, respectively; mononucleotides are excluded. A total of 5,180 SSRs, with 4,437 potentially unique SSRs, were represented in approximately 85.6% of the genes (S6 Fig). The minimum and maximum length of the repeats was 12 and 25 respectively, and the average length was 17 nucleotides. The most common motifs are listed in Table 3. A complete list of SSRs and corresponding information are provided in S5 Table. We also mined potential SNPs in CS and CT lines (25°C) using default parameters of software GATK. A massive collection of CS and CT SNPs based on the 25°C libraries included 29,106 and 27,685 genes, respectively, 19,588 were common genes were found with SNPs. Within the detected SNPs, the 63 and 62% of transition (A/G, C/T) type were found in CS and CT lines, respectively. Transversion (A/C, A/T, G/C, G/T) SNP ratios in CS and CT lines (36 and 37%, respectively) can be used to measure the genetic distance between the lines (see details in S6 Table). Transition polymorphism was more frequent than transversion, consistent with the nature of these changes [62].

**Table 3:** Summary of SSRs identified in the assembled genes from transcriptome.

S. No	Repeats	Min-max length	Most common motif	Genes
1.	Dinucleotide (AA≥6)	12–24	AT (447), GT (378)	1,465
2.	Tri nucleotide (AAA≥4)	12–24	AAG (305)	3,253
3.	Tetra nucleotide (AAAA≥4)	16–24	ATTT (63)	370
4.	Penta nucleotide (AAAAA≥4)	20–25	AAAAT (12)	59
5.	Hexa nucleotide (AAAAAA≥4)	24	AAAAAG, AAAACC, AACCTC (2)	33

## Conclusions

In this study, we used high-throughput sequencing data to characterize the transcriptomes of contrasting genotypes in *Allium cepa* (bulb onion), a species for which little genomic is information available. Comparative analysis of expression profiles revealed a large number of cold and freezing responsive differentially expressed genes. Pathway analysis of the 491 enriched DEGs, revealed major metabolism involved during freezing conditions. Re-validation with qRT- PCR demonstrated a high reliability (99%) of candidate cold and freezing-related genes. This study provides a platform for further functional genomic research on *Allium* spp., including genomic resources such as molecular markers (SSRs and SNPs) for cold and freezing stress responsiveness and will support molecular breeding and gene cloning in *Allium* spp.

## Supporting Information

S1 Fig Electrolyte leakage analysis of contrasting genotypes in *Allium cepa* L. Means followed by dissimilar letters are significantly different based on LSD test at P = 0.05 level.
(TIFF)

S2 Fig Species-wise classification based on the nr (BLASTX) hits of the genes identified in the *de novo* assembled genes. (TIFF)

S3 Fig Venn diagram comparison of CS and CT lines showing, DEGs up- and down-regulated in the respective treatments. (TIFF)

S4 Fig Volcano plots of CT and CS lines at 25 vs -5°C. Red and blue dots are indicate as up- and down regulated genes respectively. B. Venn diagram of DEG in CT and CS line at 25 vs -5°C were unique and commonly expressed.
(TIFF)

S5 Fig GO classification of DEGs in CS vs. CS and CT vs. CT. Histogram of the GO annotation and enrichment analysis was generated by the BGI WEGO software. The genes were grouped into three main GO categories: Cellular component (CC), Molecular function (MF), Biological Process (BP). The right Y- axis indicates the number of genes in a category. The left Y-axis indicates the percentage in a specific category. One gene could be assigned with more than one GO term. Color indicates GO enrichment (significantly enriched GO terms with Bonferroni corrected p-value <0.05) and GO terms in all genes.
(TIFF)

S6 Fig Venn diagram of SSR motifs (di, tri, tetra, penta and hexa nucleotide) were predicted by *in silico* analysis. (TIFF)

S1 Table Validation of candidate genes with qPCR analysis using CT libraries of cold and freezing stresses. (XLSX)

S2 Table Classification of pathways were mapped under KEGG database using assembled genes of transcriptome. (XLSX)

S3 Table The ID's and FPKM of each gene exhibiting difference among all the libraries. (XLSX)

S4 Table Combined genes expressed at freezing temperature in CT and CS lines. (XLSX)

S5 Table Complete list of SSRs marker from assembled genes of transcriptomes. (XLSX)

S6 Table The number of SNP transistion and transversion observed in *A*. *cepa* L. transcriptomes. (XLSX)
