# Supplementary figures and images for: De Novo Assembly and Transcriptome Analysis of Bulb Onion (Allium cepa L.) during Cold Acclimation Using Contrasting Genotypes
Source: PLoS One. 2016 Sep 14;11(9):e0161987. doi: 10.1371/journal.pone.0161987 (PMC5023330; doi:10.1371/journal.pone.0161987)

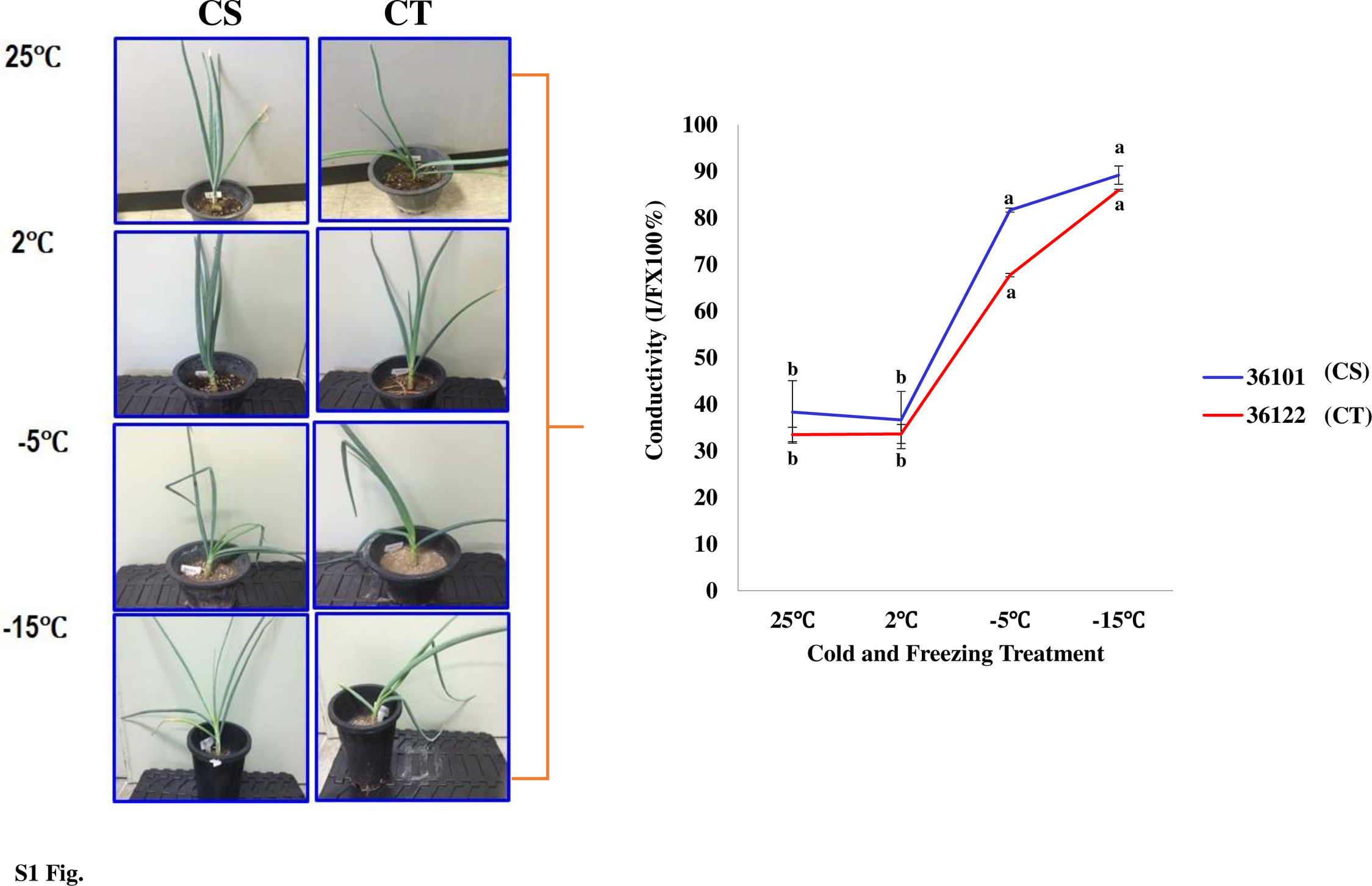

Supplement: S1 Fig — Means followed by dissimilar letters are significantly different based on LSD test at P = 0.05 level. (TIFF) [file pone.0161987.s001.tiff]

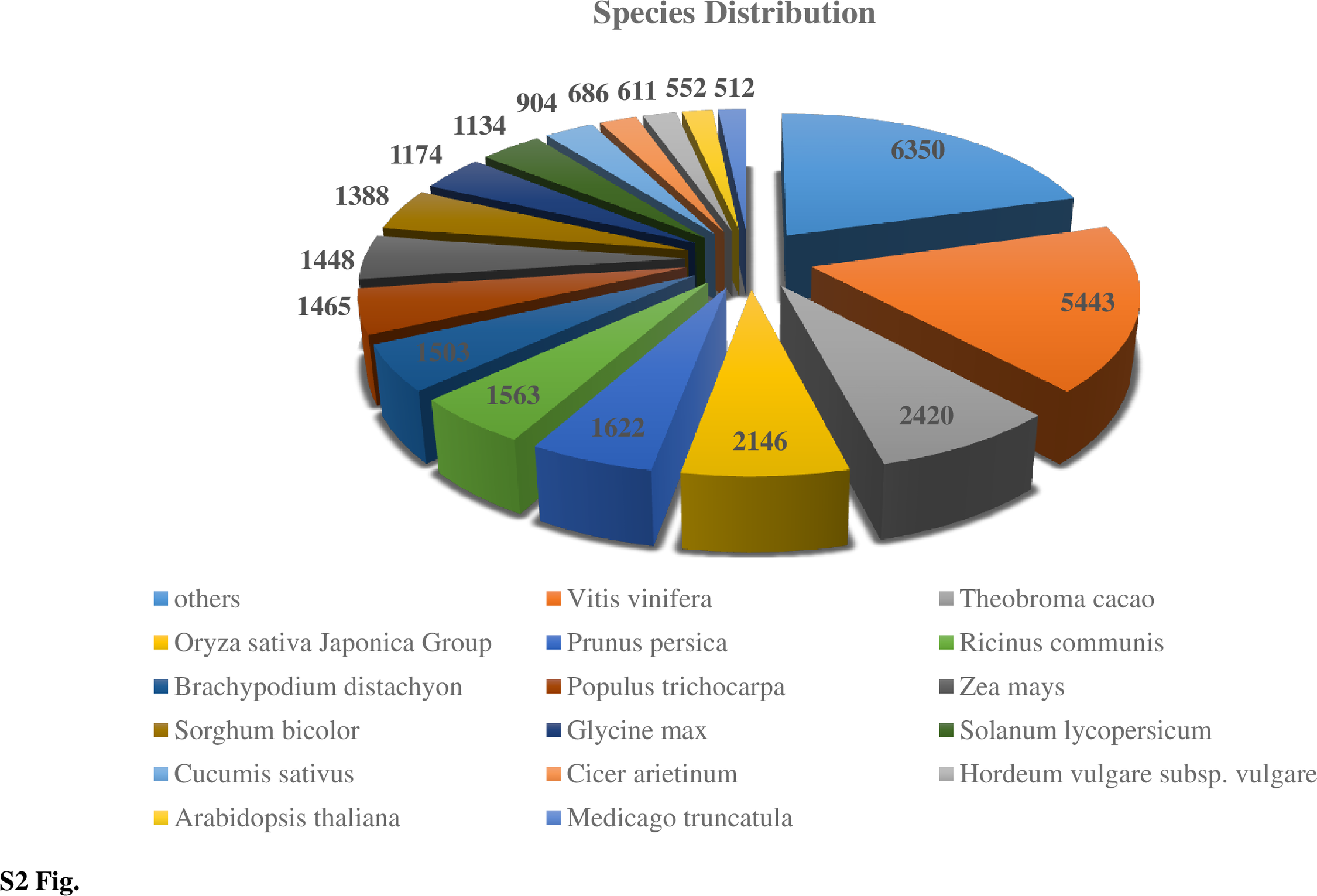

Supplement: S2 Fig — (TIFF) [file pone.0161987.s002.tiff]

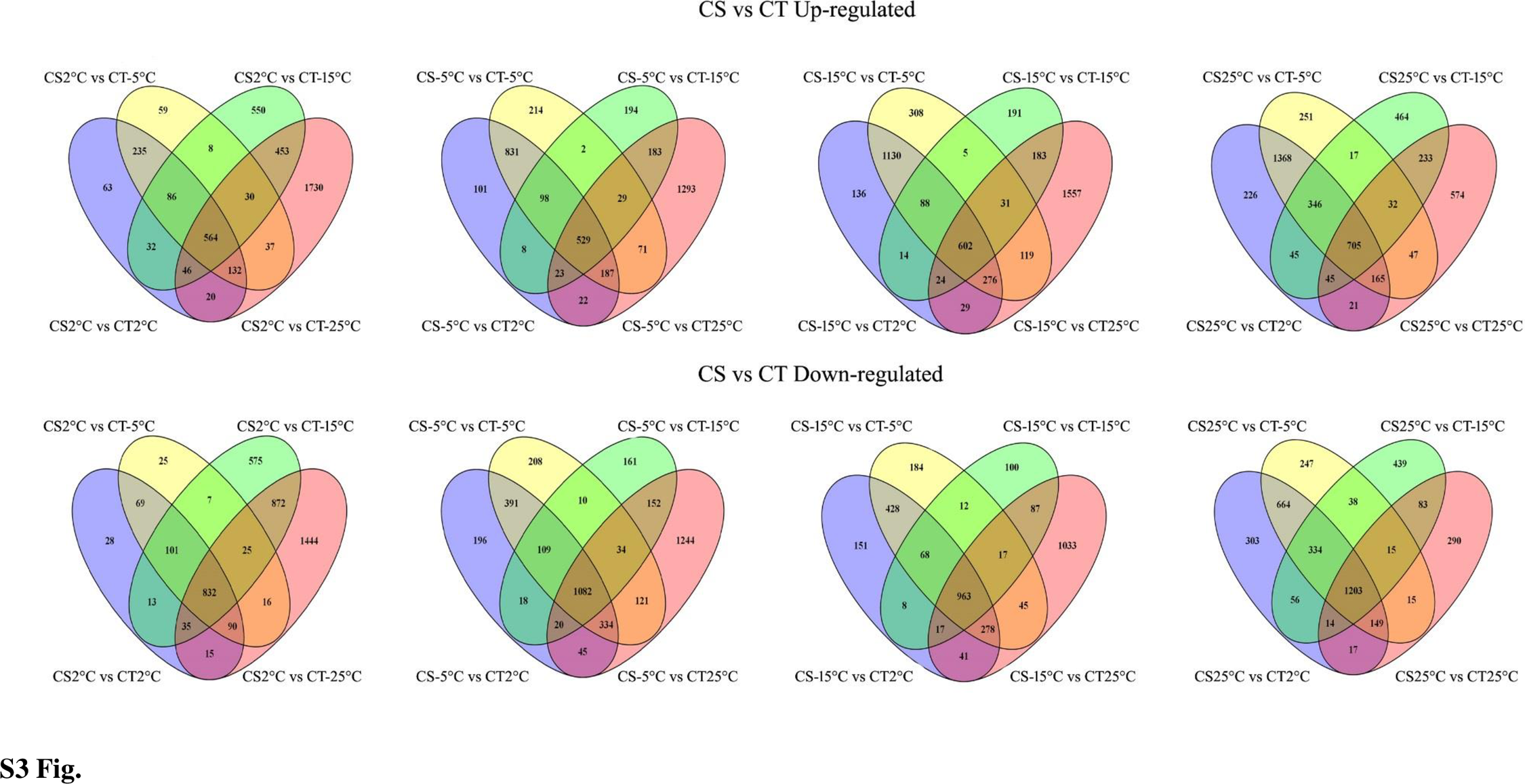

Supplement: S3 Fig — (TIFF) [file pone.0161987.s003.tiff]

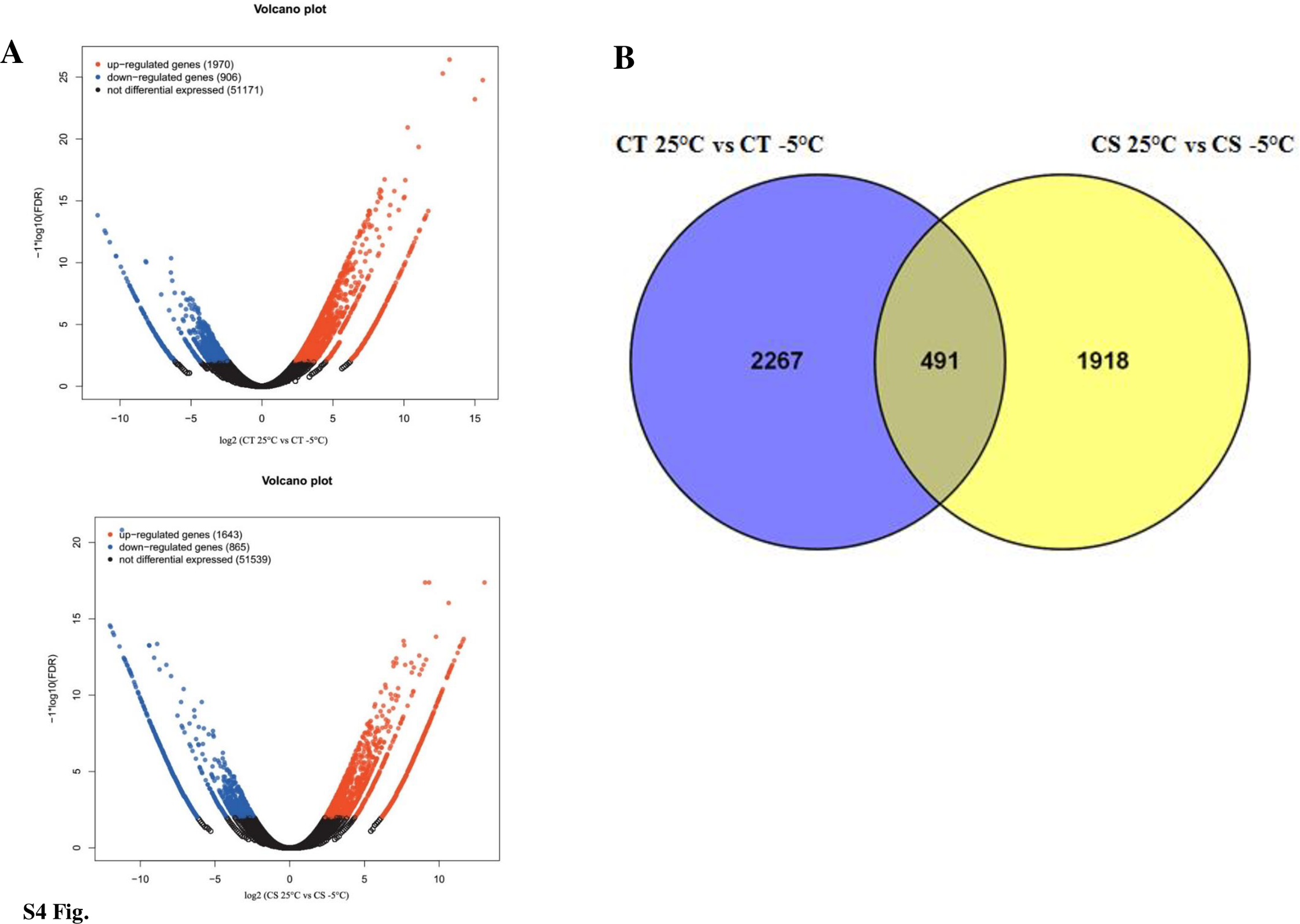

Supplement: S4 Fig — Red and blue dots are indicate as up- and down regulated genes respectively. B. Venn diagram of DEG in CT and CS line at 25 vs -5°C were unique and commonly expressed. (TIFF) [file pone.0161987.s004.tiff]

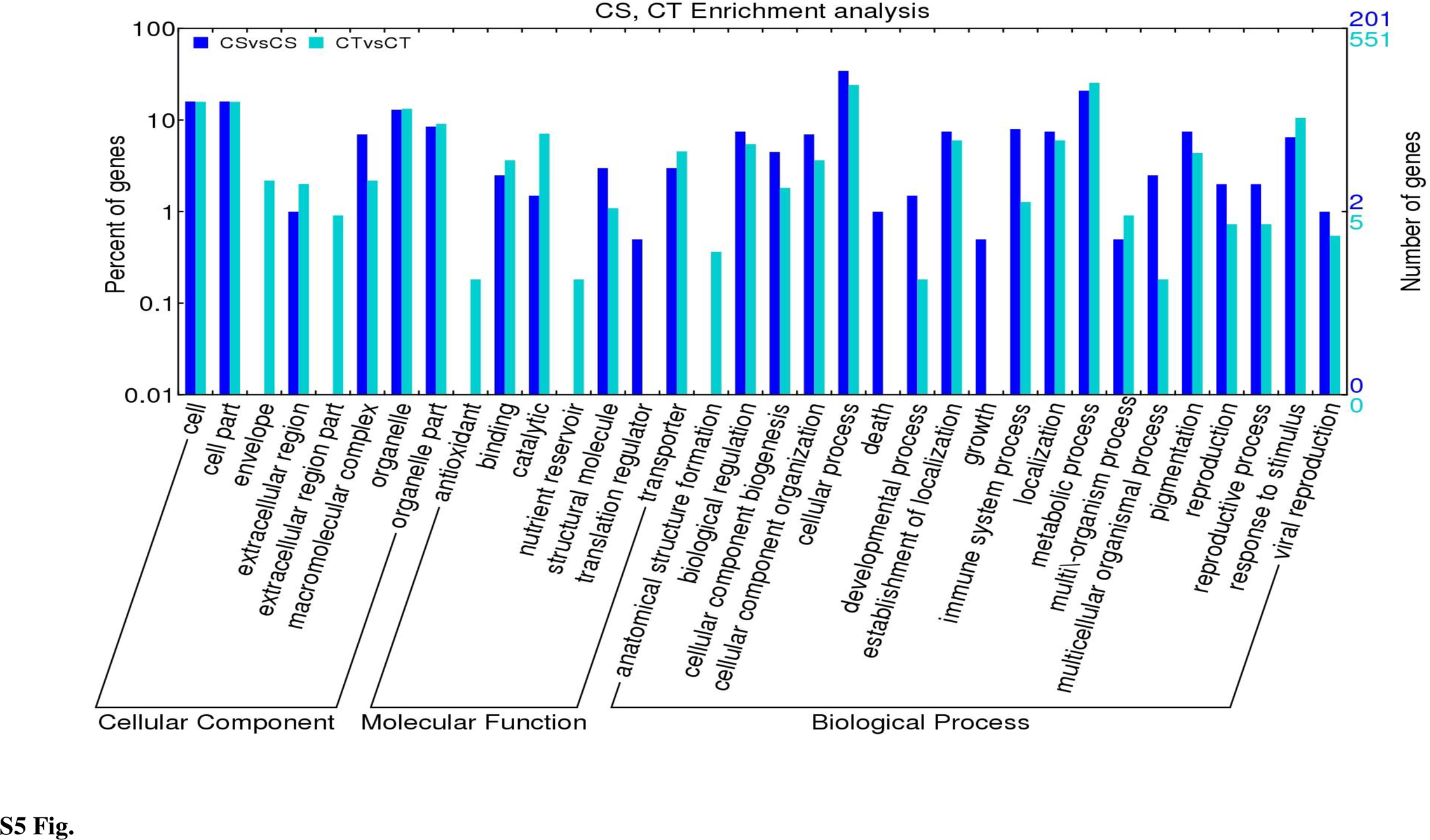

Supplement: S5 Fig — Histogram of the GO annotation and enrichment analysis was generated by the BGI WEGO software. The genes were grouped into three main GO categories: Cellular component (CC), Molecular function (MF), Biological Process (BP). The right Y- axis indicates the number of genes in a category. The left Y-axis indicates the percentage in a specific category. One gene could be assigned with more than one GO term. Color indicates GO enrichment (significantly enriched GO terms with Bonferroni corrected p-value <0.05) and GO terms in all genes. (TIFF) [file pone.0161987.s005.tiff]

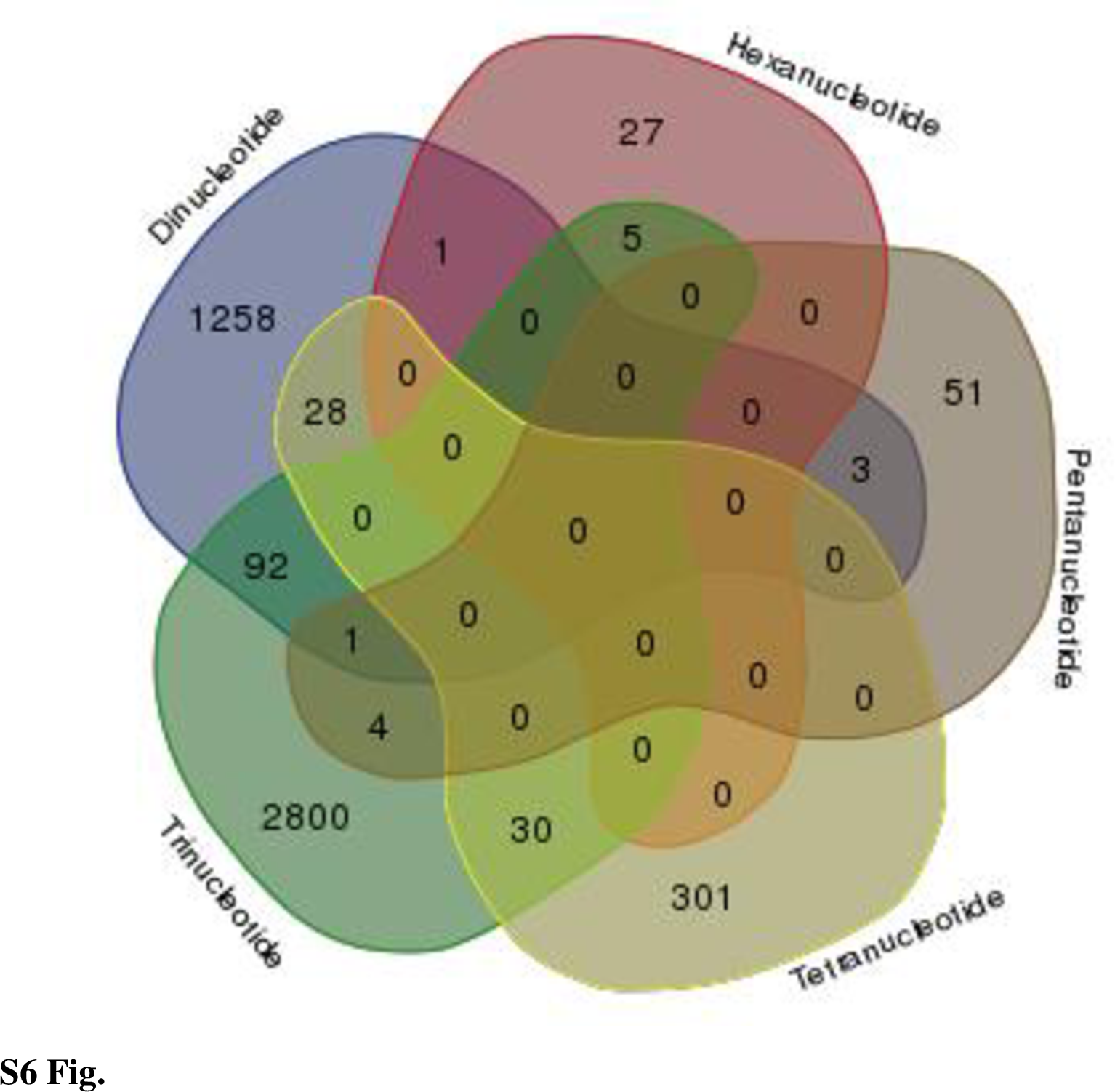

Supplement: S6 Fig — (TIFF) [file pone.0161987.s006.tiff]
